# An Unusual Association: Collapsing Glomerulonephritis in a Patient With Type 1 Diabetes

**DOI:** 10.7759/cureus.89622

**Published:** 2025-08-08

**Authors:** Zaza Aladashvili, Luka Beridze, Elene Zaalishvili, Luka Bichinashvili, Anna Shamanadze

**Affiliations:** 1 Faculty of Medicine, Tbilisi State Medical University, Tbilisi, GEO; 2 Department of Internal Medicine, Petre Shotadze Tbilisi Medical Academy, Tbilisi, GEO; 3 Department of Internal Medicine, David Tvildiani Medical University, Tbilisi, GEO; 4 Department of Internal Medicine, Tbilisi State Medical University, Tbilisi, GEO; 5 Department of Nephrology, Georgian American University, Tbilisi, GEO

**Keywords:** collapsing glomerulonephritis, immunofluorescence, kidney biopsy, kidney failure, type 1 diabetes mellitus (t1dm)

## Abstract

This case report describes a 38-year-old female patient with type 1 diabetes who developed collapsing-type glomerulonephritis (CTGN), a rare but severe kidney injury. The patient presented with nephrotic syndrome symptoms, including edema and hypertension. Laboratory tests showed significant proteinuria with normal serum creatinine and glomerular filtration rate. A renal biopsy confirmed CTGN, marked by podocyte injury and glomerular capillary collapse.

Given the patient's type 1 diabetes, the potential link between hyperglycemia, metabolic disturbances, and CTGN pathogenesis was explored. Treatment focused on stringent glycemic control, without immunosuppressive therapy, resulting in reduced proteinuria. This case underscores the importance of recognizing CTGN in diabetic patients and suggests a possible multifactorial component in its development. Further research is needed to clarify the underlying pathophysiology and optimize management strategies.

## Introduction

Collapsing-type glomerulonephritis (CTGN) is a rare but severe form of kidney injury characterized by segmental or global collapse of glomerular capillaries, podocyte hypertrophy or hyperplasia, and significant tubulointerstitial disease. It is most commonly observed in African American populations and affects both men and women equally [1].

The pathogenesis of CTGN involves damage to epithelial and podocyte cells. Research into its underlying mechanisms continues to evolve, with particular interest in the role of podocyte injury, as these cells are essential for maintaining glomerular integrity [2]. Additionally, genetic factors such as APOL1 variants and environmental triggers, including viral infections, are being investigated to better understand susceptibility to CTGN in different populations [3]. Mitochondrial mutations, such as COQ2 and PDSS2, have also been linked to the disease [1].

Although the majority of CTGN cases (77%) are idiopathic, the condition has also been associated with infections such as HIV, SARS-CoV-2, and parvovirus B19. In HIV patients, CTGN is often referred to as HIV-associated nephropathy (HIVAN), which is particularly prevalent among African American populations and represents a leading cause of end-stage renal disease (ESRD) [4,5]. Nearly all patients with HIVAN exhibit collapsing features on renal biopsy, further solidifying the link between the two conditions [5].

COVID-19-associated nephropathy (COVAN) has emerged as a significant complication during the pandemic, often presenting as collapsing glomerulopathy (CG). The condition is believed to result from direct viral injury to podocytes and is more prevalent in individuals with high-risk APOL1 genotypes [3,6].

Parvovirus B19 has also been implicated in CTGN, with mechanisms involving direct cytopathic effects on renal cells and the formation of immune complexes that contribute to glomerular injury [7]. Beyond infections, certain medications, including pamidronate, have been identified as potential causes of CTGN [8]. Other underlying conditions, such as systemic lupus erythematosus (SLE), diabetes, IgA nephropathy, and autoimmune diseases, have also been associated with its development [9].

Considered a variant of focal segmental glomerulosclerosis (FSGS), CTGN tends to progress more rapidly to end-stage renal disease (ESRD). Patients typically present with severe proteinuria, hypoalbuminemia, and large echogenic kidneys on ultrasound. Diagnosis is confirmed through renal biopsy, with the Columbia classification requiring at least one capillary loop obliteration and podocyte hypertrophy for a definitive diagnosis [5].

This case report describes a Caucasian female with type 1 diabetes diagnosed with collapsing glomerulopathy. It outlines the clinical progression and management, contributing to the limited literature on this rare and rapidly progressive renal condition.

## Case presentation

We present a case of a 38-year-old Caucasian woman with a history of type 1 diabetes since the age of 13 and hypertension, for which she has been receiving insulin therapy and lisinopril, respectively. In February 2023, she began experiencing mild, symptomatic lower limb edema. By May, she decided to consult the nephrology department. Prior to the visit, she had undergone several laboratory tests. Her creatinine clearance was 68 mL/minute (normal range: 49-90 mL/minute), estimated glomerular filtration rate (eGFR) was 101 mL/minute/1.73 m^2^ (normal range: 75-133 mL/minute/1.73m2), and her albumin level was 2.3 g/L (normal range: 3.7-5.0 g/L). Lipid panels showed increased levels of total, low-density lipoprotein (LDL), and high-density lipoprotein (HDL) cholesterol, and triglycerides. Her 24-hour protein loss was 9 g, and urinalysis did not reveal hematuria (Table 1).

**Table 1:** Laboratory values during admission eGFR: estimated glomerular filtration rate, LDL: low-density lipoprotein, HDL: high-density lipoprotein

Test	Patient's values	Reference range
Creatinine clearance	68 mL/minute	49-90 mL/minute
eGFR	101 mL/minute/1.73 m^2^	75-133 mL/minute/1.73 m^2^
Albumin	2.3 g/dL	3.7-5.0 g/dL
Total cholesterol	558 mg/dL	<200 mg/dL
LDL cholesterol	401 mg/dL	<160 mg/dL
HDL cholesterol	61 mg/dL	40-60 mg/dL
Triglycerides	470 mg/dL	<150 mg/dL

Her diabetes control was suboptimal, with the patient reporting elevated blood glucose levels, which were confirmed by testing during her visit.

**Figure 1:**
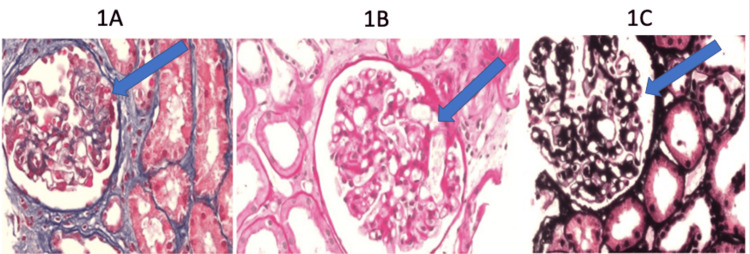
Glomerular biopsy analyzed with light microscopy and stained with Masson trichrome, PAS, and Jones Silver A: Masson's trichrome stain: A total of 29 glomeruli are observed, of which four are globally sclerosed, four are focally and segmentally sclerosed at the periphery, and one glomerulus exhibits peripheral segmental collapse with podocyte hypertrophy. B: PAS stain: A total of 29 glomeruli are observed, of which four are globally sclerosed, four are focally and segmentally sclerosed at the periphery, and one glomerulus exhibits peripheral segmental collapse with podocyte hypertrophy. C: Jones Silver stain: A total of 29 glomeruli are observed, of which four are globally sclerosed, four are focally and segmentally sclerosed at the periphery, and one glomerulus exhibits peripheral segmental collapse with podocyte hypertrophy. PAS: periodic acid-Schiff

Given her clinical presentation and laboratory results, her primary care physician decided to proceed with a kidney biopsy and additional serologic testing. The biopsy was examined using light microscopy with hematoxylin and eosin (H&E), periodic acid-Schiff (PAS), Jones Silver, and Masson trichrome stains. Of the 29 glomeruli observed, four were globally sclerosed, one exhibited collapsing glomerulopathy, and four showed focal segmental glomerulosclerosis (FSGS) with peripheral localization (Figure 1). In line with the Columbia classification of focal segmental glomerulosclerosis (FSGS), the presence of capillary loop obliteration and bordered podocyte hypertrophy confirms a diagnosis of collapsing glomerulopathy, which is considered a subtype of FSGS.

**Figure 2:**
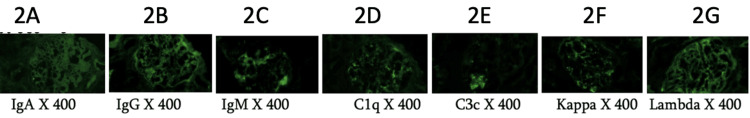
Immunofluorescence analysis with staining for IgA, IgG, IgM, C1q, C3c, and kappa and lambda light chains A, B, D, F, and G: The immunofluorescence analysis shows a light chain involvement expression of 2+. C and E: The immunofluorescence analysis shows a light chain involvement expression of 3+.

An immunofluorescence study revealed increased expression of IgM and C3c, supporting the diagnosis of collapsing-type glomerulonephritis (CTGN), a form of FSGS (Figure 2).

Further serology tests showed normal creatinine (73.26 μmol/L) and an eGFR of 91 mL/minute/1.73 m^2^. However, albumin was decreased to 2.04 g/L, and total protein was 6.12 g/L. Additionally, lipid levels were still elevated, including total cholesterol, triglycerides, and LDL (Table 2).

**Table 2:** Laboratory test results after the kidney biopsy LDL: low-density lipoprotein, HDL: high-density lipoprotein, eGFR: estimated glomerular filtration rate, ANA: antinuclear antibody, dsDNA: double-stranded DNA, HIV: human immunodeficiency virus

Test	Patient's values	Reference range
Albumin	2.04 g/dL	3.7-5.0 g/dL
Total protein	6.12 g/dL	6.40-8.20 g/dL
Atherogenic index	0.37	<0.21
Total cholesterol	308.08 mg/dL	<200 mg/dL
LDL cholesterol	174.50 mg/dL	<160 mg/dL
HDL cholesterol	56.43 mg/dL	40-60 mg/dL
Triglycerides	301.83 mg/dL	<150 mg/dL
Creatinine clearance	73.26 mL/minute	49-90 mL/minute
eGFR	91 mL/minute/1.73 m^2^	75-133 mL/minute/1.73 m^2^
ANA IgG	0.13 EU/mL	0-0.9 EU/mL
Anti-dsDNA antibodies	2.45 IU/mL	0-20 IU/mL
HIV fast test	Negative	Negative

The nephrology team conducted further diagnostic tests, including HIV, ANA IgG, and anti-dsDNA, to evaluate possible underlying causes, all of which returned negative. Given the patient's history of poorly controlled type 1 diabetes, along with metabolic disturbances such as hyperglycemia and dyslipidemia, her collapsing glomerulopathy was most likely attributed to these diabetic-related factors.

The primary care physician initiated treatment focusing on dietary changes, specifically recommending a low-sodium diet. For edema and fluid overload, the patient was prescribed a loop diuretic (torsemide) 50 mg, a potassium-sparing diuretic (spironolactone) 50 mg, and a thiazide diuretic (hydrochlorothiazide) 25 mg for hypertension. Additionally, albumin infusions were administered twice to restore her level to baseline.

The treatment plan significantly improved the patient's health status: her glucose levels normalized, edema subsided, and her overall well-being improved. Subsequent testing revealed normal creatinine levels but 2+ protein in her urinalysis. Despite this, she reported feeling well. However, she did not undergo the recommended 24-hour urine collection and was later lost to follow-up.

## Discussion

Collapsing-type glomerulonephritis (CTGN) is a rare but aggressive renal pathology that can rapidly progress to end-stage renal disease. It is most commonly associated with viral infections such as HIV, parvovirus B19, and SARS-CoV-2, as well as genetic predisposition and autoimmune diseases [10]. In this case, common secondary causes were thoroughly investigated; tests for HIV, ANA, and anti-dsDNA were performed and returned negative. The absence of these classical etiologies suggests that other factors may be involved. Notably, the patient had a long-standing history of poorly controlled type 1 diabetes, raising the possibility that chronic metabolic disturbances may play a contributory role in the pathogenesis of CTGN. This case emphasizes the need to consider CTGN in diabetic patients with unexplained nephrotic-range proteinuria, as early diagnosis and intervention may alter the disease trajectory.

Some researchers classify CTGN as a severe phenotype of focal segmental glomerulosclerosis (FSGS), where additional stressors lead to a more aggressive and rapidly progressive form of kidney disease. Some studies suggest that metabolic disturbances, inflammation, and genetic susceptibility can act as a "second hit" in FSGS, pushing it toward a collapsing glomerulopathy phenotype [11]. In this case, the patient's poorly controlled diabetes, significant proteinuria, and dyslipidemia likely served as contributing factors, driving her disease toward CTGN rather than classical FSGS or diabetic nephropathy.

From a therapeutic standpoint, this case reinforces the importance of individualized treatment strategies. While corticosteroids are often used to manage primary FSGS, their effectiveness in CTGN remains uncertain, particularly in patients with underlying metabolic disease. Given the patient's poor glycemic control, the medical team opted to avoid steroids to prevent further metabolic complications. Instead, treatment focused on strict glucose regulation, renin-angiotensin-aldosterone system (RAAS) inhibition, and diuretic therapy. The patient's clinical improvement suggests that, in certain cases, targeting metabolic dysregulation may be just as crucial as immunosuppression in halting disease progression [12]. The 2021 Kidney Disease: Improving Global Outcomes (KDIGO) guidelines provide a structured approach to CTGN management. While the overall prognosis remains poor, early diagnosis and appropriate treatment can lead to remission, particularly in patients with severe proteinuria and low eGFR [13].

This case expands the differential diagnosis of nephrotic syndrome in diabetic patients and raises important implications for understanding podocyte biology in the context of metabolic disease. It also challenges the assumption that all diabetic patients with proteinuria have diabetic nephropathy or that all FSGS cases share the same pathophysiology [14]. Recognizing these distinctions is essential, as delayed interventions, such as postponing a kidney biopsy or initiating inappropriate treatment, can lead to worse outcomes. This case underscores the need to explore whether metabolic dysfunction alone can trigger collapsing glomerulopathy. This question could enhance our understanding of podocyte injury and its broader implications in kidney disease.

## Conclusions

This case emphasizes the importance of recognizing CTGN in diabetic patients with nephrotic-range proteinuria, as metabolic stressors may contribute to its development. Accurate diagnosis through biopsy is crucial to avoid misclassification and inappropriate treatment. In cases with metabolic dysfunction, strict glycemic control may be as vital as immunosuppression in managing disease progression.
